# Deregulation of Metalloproteinase Expression in Gray Horse Melanoma Ex Vivo and In Vitro

**DOI:** 10.3390/cells13110956

**Published:** 2024-05-31

**Authors:** Daniela M. Brodesser, Stefan Kummer, Julia A. Eichberger, Karin Schlangen, Annunziata Corteggio, Giuseppe Borzacchiello, Christof A. Bertram, Sabine Brandt, Barbara Pratscher

**Affiliations:** 1 Research Group Oncology (RGO), Centre for Equine Health and Research, Department for Small Animals and Horses, University of Veterinary Medicine, Veterinaerplatz 1, 1210 Vienna, Austria; daniela.brodesser@vetmeduni.ac.at (D.M.B.); julia@eichberger.me (J.A.E.); 2 VetImaging, VetCore Facility, University of Veterinary Medicine, Veterinaerplatz 1, 1210 Vienna, Austria; stefan.kummer@vetmeduni.ac.at; 3 Section for Biosimulation and Bioinformatics, Centre for Medical Statistics, Informatics and Intelligent Systems, Medical University of Vienna (MUV), Waehringer Guertel 18-20, 1090 Vienna, Austria; karinschlangen@gmx.net; 4 Institute of Biochemistry and Cell Biology (IBBC), National Research Council (CNR), Via Pietro Castellino 111, 80131 Naples, Italy; annunziata.corteggio@ibbc.cnr.it; 5 Department of Veterinary Medicine and Animal Productions, University of Naples Federico II, Corso Umberto I 40, 80138 Naples, Italy; borzacch@unina.it; 6 Institute of Pathology, Department of Pathobiology, University of Veterinary Medicine, Veterinaerplatz 1, 1210 Vienna, Austria; christof.bertram@vetmeduni.ac.at; 7 Division of Small Animal Internal Medicine, Department for Small Animals and Horses, University of Veterinary Medicine, Veterinaerplatz 1, 1210 Vienna, Austria; barbara.pratscher@vetmeduni.ac.at

**Keywords:** gray horse melanoma, epithelial–mesenchymal transition, matrix metalloproteinases, MMP1, expression, primary melanoma cells

## Abstract

The ability of human melanoma cells to switch from an epithelial to a mesenchymal phenotype contributes to the metastatic potential of disease. Metalloproteinases (MPs) are crucially involved in this process by promoting the detachment of tumor cells from the primary lesion and their migration to the vasculature. In gray horse melanoma, epithelial–mesenchymal transition (EMT) is poorly understood, prompting us to address MP expression in lesions versus intact skin by transcriptome analyses and the immunofluorescence staining (IF) of gray horse tumor tissue and primary melanoma cells. RNAseq revealed the deregulation of several MPs in gray horse melanoma and, notably, a 125-fold upregulation of matrix metalloproteinase 1 (MMP1) that was further confirmed by RT-qPCR from additional tumor material. The IF staining of melanoma tissue versus intact skin for MMP1 and tumor marker S100 revealed MMP1 expression in all lesions. The co-expression of S100 was observed at different extents, with some tumors scoring S100-negative. The IF staining of primary tumor cells explanted from the tumors for MMP1 showed that the metalloproteinase is uniformly expressed in the cytoplasm of 100% of tumor cells. Overall, the presented data point to MP expression being deregulated in gray horse melanoma, and suggest that MMP1 has an active role in gray horse melanoma by driving EMT-mediated tumor cell dissemination via the degradation of the extracellular matrix. Whilst S100 is considered a reliable tumor marker in human MM, gray horse melanomas do not seem to regularly express this protein.

## 1. Introduction

Recent developments and approvals of innovative tumor therapeutics have significantly changed the landscape of cancer management. However, the therapy of malignant melanoma (MM) continues to be challenging or futile in human and animal patients. In human MM (hMM), therapy resistance and metastasis are also due to the high plasticity of tumor cells [1]. Epithelial-type melanoma cells can switch to a mesenchymal phenotype driving extracellular matrix (ECM) degradation, cell motility, invasion, and metastasis [2]. Melanoma cells can also acquire an endothelial phenotype that builds up an alternative system of microvessels termed “vasculogenic mimicry” that facilitates metastases. Importantly, epithelial tumor cells can turn into cancer stem cells (CSCs). This phenotype confers multidrug resistance (MDR) and is associated with disease progression, metastasis, and hence poor prognosis [3].

The epithelial–mesenchymal transition (EMT) of tumor cells is mediated by transcription factors repressing E-cadherin and enhancing the expression of mesenchymal proteins [4]. The latter include metalloproteinases (MPs) that have a major role in the degradation and remodeling of the ECM and basement membrane, thus paving the way for tumor cell detachment from the primary lesion and migration to blood and lymphatic vessels [5]. According to their substrate specificity and structural features, MPs are classified as matrix metalloproteinases (MMPs), a disintegrin and metalloproteinases (ADAMs), and ADAMs with thrombospondin motifs (ADAMTS) [6].

Under physiological conditions, MPs assure tissue integrity, and their activity is finely tuned by various growth factors, cytokines, and chemokines, as well as endogenous tissue inhibitors of metalloproteinases (TIMPs) [7]. In human MM (hMM) and other human cancers, the delicate balance between MPs and their inhibitors is disturbed, leading to the overexpression of MPs with pro-tumor functions, whilst MPs with anti-tumor properties remain inactive. In addition, the concurrent expression of specific adhesion molecules is thought to help position the active proteases at the tumor’s invasive front [5]. In hMM, the radial growth phase (RGP; early tumor stage) and the vertical growth phase (VGP) are each characterized by specific MP expression signatures as summarized in Table 1 [7].

**Figure 1:**
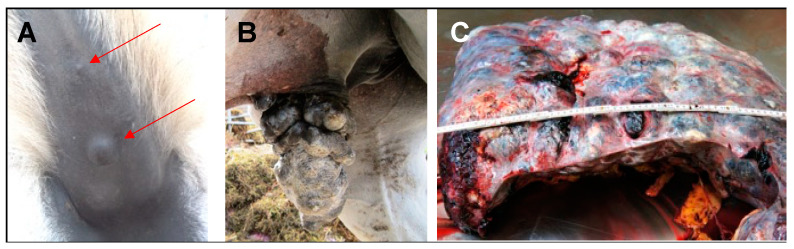
Gray horse melanoma. (**A**): Two quiescent, encapsulated nodular melanomas (red arrows) under the tail root of 18-year-old Welsh Mountain pony (LOD; Table 2). (**B**): Rapidly growing melanomas on the prepuce of a 13-year-old Trakehner gelding (DIM; Table 2). (**C**): Spleen metastases in 12-year-old Warmblood mare (ELL; Table 2), leading to lethal organ disruption. The spleen weighted 34 kg at necropsy; further metastases affected the liver, colon, and heart. All metastases originated from a cutaneous melanoma affecting the perianal region.

Melanoma not only affects humans but also occurs in various animal species as reviewed recently [8]. In horses, up to 15% of skin neoplasms are melanocytic. As a unique feature, the incidence and pathological behavior of equine melanoma depend on the coat color. In non-gray horses, disease is rare and usually malignant, with early metastasis representing a typical event. In gray horses, however, disease is commonly observed, with up to 80% of animals older than 15 years exhibiting melanocytic lesions. These lesions preferentially develop underneath the tail, the anogenital region (Figure 1), the lips, eyelids, and parotid region, but also at other cutaneous and mucosal sites [9].

Interestingly, gray horse melanomas often remain quiescent or grow slowly over several years before they progress and metastasize [9,10] (Figure 1). The deceleration of malignant growth may be due to the fact that cutaneous-type lesions are often encapsulated and thus poorly vascularized [11]. The biological mechanisms underlying this phenomenon are still unclear. It is thinkable that tumor encapsulation has emerged as an evolutionary strategy to mitigate the effect of the 4.6-kbp duplication in synthaxin-17 intron 6, which concurrently accounts for the gray phenotype and the predisposition for melanoma [12].

Whilst the contribution of MPs to hMM progression and metastasis is well documented, there is no information on the role of MPs in equine melanoma so far. This lack of knowledge led us to assess MP expression in gray horse melanoma in a multiple-step approach. We first analyzed the MP transcription pattern in lesions versus intact skin of gray horses using next-generation RNA sequencing (RNAseq). The thereby determined 125-fold upregulation of MMP1 in melanoma tissue was subsequently validated by RT-qPCR from further ex vivo samples. In the next step, we established equine primary melanoma cells from fresh tumor material and comparatively assessed the expression of MMP1 in tumor tissue and primary cells derived therefrom by immunofluorescence staining (IF). Overall, we show that the MT expression signatures of gray horse and human cutaneous melanoma differ significantly, with MMP1 being overexpressed in tumor tissue and primary melanoma cells on the mRNA and protein level.

## 2. Materials and Methods

### 2.1. Sample Material

The study involved nine gray horses affected by cutaneous melanoma and two tumor-free gray horses. All horse and sample specifications, as well as the type of experiments to which the samples were subjected, are provided in Table 2.

Tumor specimens were obtained with the owner’s written consent and in accordance with institutional guidelines allowing tissue excised for diagnostic or therapeutic reasons to be used for scientific purposes. Intact skin was uniformly collected from the horses’ necks with the approval of the institutional ethics committee and the Federal Ministry of Science and Research (BMWF; license Nr.: GZ 68.205/0102-II/3b/2013) under local anesthesia using a 4 mm biopsy punch.

The lesions were diagnosed as equine melanoma by clinical examination. The diagnosis was confirmed by a histopathological assessment of the tumor sections. To this aim, fresh tumor material was fixed in 4% neutral buffered formalin for 24 h, gradually dehydrated and embedded in paraffin. Then, 2.5 µm tumor sections were prepared using the rotation-microtome Microm HM355S (Thermo Fisher Scientific, Waltham, MA, USA). The sections were dried overnight at 37 °C, then deparaffinized in xylene, rehydrated in ethanol at decreasing concentrations, and subjected to hematoxylin and eosin (H&E) staining. Subsequently, the sections were analyzed by microscopy using a Leitz Dialux 20 microscope (Leica Microsystems, Wetzlar, Germany).

### 2.2. Next-Generation RNA Sequencing (RNAseq)

In the first step, MP transcription in cutaneous gray horse melanoma versus intact skin was addressed by RNAseq. To this aim, a series of tumor and intact skin aliquots kept in 500 µL of TRI Reagent^®^ (Sigma-Aldrich, Vienna, Austria) were homogenized using an IKA Ultra-Turrax T8 Disperser (IKA-Werke, Staufen, Germany). Following the addition of 350 µL of chloroform per tube and vigorous shaking, the samples were incubated for 10 min at room temperature, and then centrifuged at 12,000× *g* for 15 min at 4 °C. The upper aqueous phases were transferred to 1.5 mL Eppendorf tubes and supplemented with 250 µL of ice-cold isopropanol. The tubes were shaken vigorously and subsequently centrifuged at 12,000× *g* for 45 min at 4 °C. Then, the supernatants were discarded and the pellets were washed with 500 µL of 75% ethanol, air-dried, and resuspended in 20 µL of sterile water. Subsequent DNase digestion was carried out in RNeasy columns (Qiagen, Hilden, Germany) according to the instructions of the manufacturer. The obtained DNA-free RNA was eluted with 22 µL of sterile water into 1.5 mL Eppendorf tubes, each containing 1 µL of the RNase inhibitor (RNaseOUT; Invitrogen, Thermo Fisher Scientific). To remove melanin remnants reflected by the yellowish color of the eluates, an additional cleanup step was performed using an RNeasy Mini kit (Qiagen) according to the instructions of the manufacturer.

RNA concentrations were assessed by photometry (Eppendorf BioPhotometer^TM^ D30, Hamburg, Germany). RNA integrity was determined using a Bioanalyzer RNA 6000 Nano assay (Agilent Technologies, Vienna, Austria). RNA isolates with an OD 260/280 ranging between 1.8 and 2.2, an OD 260/230 > 2.0, and an RNA integrity number (RIN) > 7 (for selected samples see Table 2, “Analyses performed”) were submitted to BGI Genomics (Shenzhen, China) for RNAseq and basic bioinformatics analysis.

The obtained reads were filtered for adaptors and low-quality reads, then aligned to the whole genome Shotgun assembly EquCab2 (GenBank Assembly ID GCA_000002305.1) of the thoroughbred mare “Twilight” [13] using SOAP2, an alignment program [14]. Following further filtering steps and quality checks, uniquely mapped reads in proper pairs were mapped to genes (gene expression annotation) and calculated. For the determination of differentially transcribed genes, the expression levels of reads per kilobase per million reads (RPKM) were calculated. To correct for multiple testing, the false discovery rate method (FDR) was used [15]. Only genes revealing a ≥2-fold deregulation of expression and an FDR < 0.001 were considered as being differentially expressed (manuscript in preparation).

RNAseq analysis resulted inter alia in a 125-fold upregulation of MMP1 in melanoma tissue compared to intact skin. This finding was further validated by RT-qPCR from additional tumor and skin samples (see Table 2, “Analyses performed”).

### 2.3. MMP1 RT-qPCR

High-quality DNA-free RNA was isolated from tumor and intact skin aliquots (Table 2) as described above (RNAseq). Complementary DNA synthesis was conducted in MμlTI Ultra PCR tubes (Sorenson Bioscience, Salt Lake City, UT, USA), each containing 50 mM of Tris–HCl, pH 8.3, 75 mM of KCl, 3 mM of MgCl2, 10 mM of DTT, 2 mM of each dNTP, 200 pmol of random hexamer (Invitrogen), 20 U of a recombinant RNase inhibitor (RNaseOUT; Invitrogen), 5 μL of RNA, and 200 U of SuperScript III reverse transcriptase (+RT; Invitrogen Life Technologies, Vienna, Austria) or 1 μL of sterile water as a mock RT control (−RT) in a total volume of 20 μL. Reverse transcription was carried out by incubation at 48 °C for 1 h in a thermocycler (MJ Research MiniCyclerTM, Biozym, Hessisch Oldendorf, Germany).

Following beta-actin PCR [16] from all cDNA and control aliquots confirming successful RNA isolation, DNase digestion, and cDNA synthesis, the samples were subjected to MMP1 qPCR. Equine 40S ribosomal protein S13-like (RPS13) served as a reference housekeeping gene in the assay [17,18]. The reactions were conducted in duplicates in final 20 µL volumes containing 10 μL of qPCR TaqMan^®^ Universal PCR Master Mix (Thermo Fisher Scientific), 1 µL of MMP1 Assay Mix FAM (Assay ID: Ec03468020_m1: Thermo Fisher Scientific) or RPS13 Assay Mix VIC (5′RPS13: 5′-GTGCCCACTTGGCTGAAGTT-3′; 3′RSP13: 5′-TCTTGGCCAGCTTGTAGATCTG-3′; 5′-Probe: VIC-CGTCTGACGACGACGTGAG; Applied Biosystems, Life Technology, Vienna, Austria), 4 µL of sterile water, and 5 µL of template (cDNA or -RT-control, and sterile water as the no-template control). The thermal cycling program consisted of 50 °C for 2 min and 95 °C for 10 min, followed by 40 cycles at 95 °C for 15 s and 60 °C for 1 min. The amplification reaction was performed in a StepOneTM Real-Time PCR cycler (Thermo Fisher Scientific). Quantitative data analysis was carried out using QuantStudio Real-Time PCR Software version number 1.3 (Thermo Fisher Scientific). The fold differences in gene expression were calculated by the ΔΔCt method [19] on the basis that MMP1 and RPS13 qPCR showed analogous amplification efficiencies as determined by the qPCR from the template dilution series.

### 2.4. Establishment of Equine Primary Melanoma Cells

MMP1 overexpression in gray horse melanoma was determined by RNAseq and confirmed by RT-PCR. To be able to investigate whether MMP1 transcripts are translated into protein, and whether this protein is specifically expressed by equine tumor cells, we established primary melanoma cells from five fresh tumor samples. They were derived from DOL (initial tumor and recurrent lesion), ELL (liver metastasis), MAK, and TIM, as indicated in Table 2. Following excisional biopsy, the tumor aliquots were immediately supplied with Dulbecco’s Modified Eagle Medium (DMEM) with high glucose (4.5 g/L) and GlutaMAX™ supplemented with 10% fetal bovine serum, 1% antibiotic–antimycotic mixture (all from GIBCO Life Technologies, Carlsbad, CA, USA) and transferred to the laboratory. Then, the tumor tissue was separated from the surrounding tissue using a scalpel, shredded, and washed multiple times with Dulbecco’s phosphate-buffered saline (PBS) without magnesium and calcium (GIBCO Life Technologies). Subsequently, the tumor aliquots were digested in 2.5% Trypsin solution diluted 1:10 in Hanks balanced salt solution (HBSS; all from GIBCO Life Technologies) at 4 °C overnight, followed by incubation at 37 °C for 90 min. The digested samples were filtered and scraped through a 100 µm Cell Strainer (BD Falcon, Heidelberg, Germany), collected in FBS, and then washed repeatedly with 50 mL of PBS until the supernatants appeared clear. The obtained cells as well as EqMel were seeded in T25 flasks and incubated at 37 °C in a 5% CO_2_ atmosphere. The medium was changed twice a week and cells were split according to their density using 0.05% Trypsin-EDTA (GIBCO Life Technologies). According to their respective source, the primary cells established by us were designated as DOLmc, DOLrmc, MAKmc, TIMmc, and ELLmtc, with mc denoting melanoma cells, rmc cells from recurrent melanoma, and mtc cells from a metastasis.

Primary gray horse melanoma cells “EqMel” were kindly provided by Prof. Lubna Nasir (University of Glasgow, Scotland) and used as control cells.

### 2.5. Immunofluorescence (IF) Labeling of Tumor Sections and Cultured Melanoma Cells for MMP1

IF single- and double-labeling experiments were carried out according to established protocols [20,21].

The double-labeling of tissue sections for MMP1 and S100 involved the following: FFPE sections (2.5 µm) of lesions and intact skin from CUR, DIM, GYN, and LOR, as well as of tumors from ELL (perianal tumor and liver metastasis), DOL (initial and recurrent melanoma), MAK, and TIM were deparaffinized with xylene and gradually rehydrated in 100%, 96%, and 70% ethanol. Following the blocking of unspecific binding sites with 10% normal goat serum, sections were incubated with the rabbit polyclonal anti cattle-S100A1 antibody (diluted 1:10,000 in PBS; #Z0311, Dako, Glostrup, Denmark) at 4 °C overnight. After the incubation of the secondary anti-rabbit poly-HRP antibody (ready-to-use; BrightVision #DPVR-HRP, ImmunoLogic, Duiven, NL, USA) for 60 min, the signal was developed with an Alexa 568-labeled tyramide solution (stock solution prepared according to the datasheet with dimethyl sulfoxide and then diluted 1:100 in TRIS–HCl buffer with 0.003% H_2_O_2_; Invitrogen Tyramide Conjugates, ThermoFisher Scientific, Vienna, Austria) for 10 min.

Following the stripping of the antibodies with Tris–EDTA buffer (pH 9) and boiling for 15 min in the microwave, the sections were blocked with 10% normal goat serum and then incubated with the affinity-purified rabbit polyclonal anti human-MMP-1 antibody (N-17)-R (diluted 1:1500 in PBS; #sc-8834-R, Santa Cruz Biotechnology, Inc, Heidelberg, Germany) at 4 °C overnight. Then, the binding of this primary antibody was detected by incubation with the secondary anti-rabbit poly-HRP antibody (BrightVision) and subsequent development with Alexa 647-labeled tyramide solution. Finally, cell nuclei were counterstained with DAPI (#D9542, Sigma-Aldrich, St. Louis, MO, USA).

According to the human protein atlas (v23.proteinatlas.org), equine skin was included in the experiment as a positive control for S100 (positive for melanocytes, nerves, skeletal muscle) and MMP1 (expressed, e.g., by exocrine glandular cells). The images of the tissue sections were taken using a fluorescence microscope (Axio Imager Z2, Zeiss, Jena, Germany) equipped with a highly sensitive camera (AxioCam MR R3, Zeiss, Jena, Germany).

The single-labeling of cultured melanoma cells for MMP1 involved the following: After fixation, cells were incubated with 0.25% Triton X-100 (#X100, Sigma-Aldrich, St. Gallen, Switzerland) in PBS for 10 min to increase permeabilization. Then, the cells were incubated with Tris–EDTA buffer at pH 9 at 90 °C in a water bath, blocked with 10% goat serum and then incubated with the primary anti-MMP1-antibody as described above. MMP1 zymogen-antibody complexes were detected by incubation with the anti-rabbit poly-HRP antibody (BrightVision) and subsequent development with Alexa 647-labeled tyramide solution. Cell nuclei were counterstained with DAPI (#D9542, Sigma-Aldrich) and imaged using a confocal laser scanning microscope (CLSM 880, Zeiss).

## 3. Results

### 3.1. Histopathological Examination Reveals Typical Characteristics of Equine Melanoma

**Figure 2:**
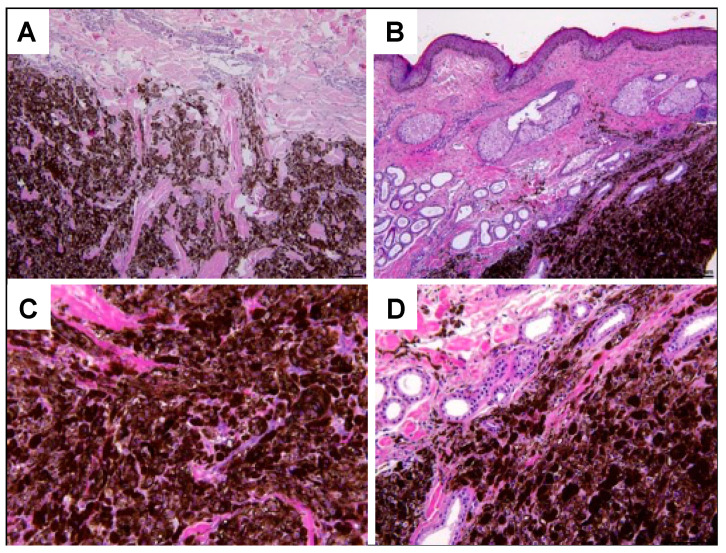
Microphotographs of HE-stained melanoma sections. (**A**,**B**): Initial tumor from DOL (Table 2) at 4× (**A**) and 10× (**B**) magnification. (**C**,**D**): Tumor from MAK (Table 2) at 4× (**C**) and 10× (**D**) magnification. Both lesions were nodular, but not encapsulated. Whilst around 50% of melanoma cells composing the lesion of horse DOL showed melanin pigmentation (**A**,**B**), virtually all melanoma cells were heavily pigmented in the tumor originating from the equine patient MAK (**C**,**D**). Images were taken using a camera-equipped Leitz Dialux 20 microscope (Leica Microsystems).

To confirm visual melanoma diagnosis, equine tumor sections were subjected to HE staining and then examined by microscopy (Figure 2). All skin lesions exhibited typical characteristics of melanocytic tumors. The lesions were nodular, commonly extending from apocrine sweat glands into the deeper dermis. Most lesions were well demarcated, with only a few melanocytes being observed in the surrounding tissue. Depending on the tumor examined, 50 to almost 100% of cells were heavily pigmented and displayed coarse melanin granules. The tumor cells exhibited a polygonal shape, and some were round or fusiform. Tumor cell nuclei contained one to multiple nucleoli and uniformly displayed moderate anisokaryosis. The HE sections of two lesions (DOL, MAK; see Table 2) exhibiting these features are exemplarily shown in Figure 2. The liver metastasis mainly consisted of jet-black round to polyhedral tumor cells (ELL meta).

### 3.2. RNAseq Reveals a Deregulation of MP Transcription in Lesional Tissue

A comparison of mean filtered, unique mapped reads in gray horse melanoma (Table 3; $\bar{x}$ mel) versus normal skin (Table 3; $\bar{x}$ skin) revealed significant differences in the transcription of MPs of various type (MMP, ADAM, ADAMTS). More than 4-fold deregulation events with mean filtered, unique mapped reads > 100, a *p*-value ≤ 0.05, and an FDR < 0.0001 are presented as the most significant findings in Table 3.

The highest difference in mean transcription levels was exhibited by MMP1 with a 125-fold upregulation in gray horse melanoma compared to intact skin. Given the limited number of RNA isolates of sufficient quality that could be subjected to RNAseq, this finding was further validated by RT-qPCR.

### 3.3. RT-qPCR Confirms the Upregulation of MMP1 Transcription in Gray Horse Melanoma

The total RNA was isolated from additional tumor samples and intact skin (Table 2), DNase digested, and subjected to MMP1 RT-qPCR, with RPS13 serving as the reference housekeeping gene. The relative quantification of MMP1 transcription in melanoma versus intact skin was carried out by the comparative ΔCT method. The obtained findings are presented in Table 4.

RT-qPCR revealed an almost 26-fold upregulation of MMP1 transcription in tumor tissue compared to intact skin.

In the case of horses DIM and LOR, tumor tissue and intact skin were available for the comparison of MMP1 transcription within each individual. In both horses, MMP1 was ±55-fold overexpressed in the lesions compared to the intact skin collected from the neck. The respective 2^−ΔΔCT^ values amounted to 55.75 (DIM) and 54.88 (LOR). 

In the same experiment, we also assessed melanoma cells derived from DOL for relative MMP1 transcription in comparison to the intact skin from this horse. With a 2^−ΔΔCT^ value of 0.48, DOL cells and intact skin exhibited similar levels of MMP1 transcription, as shown in Table 5.

### 3.4. MMP1 Is Consistently Expressed in Equine Melanoma and Metastases

**Figure 3:**
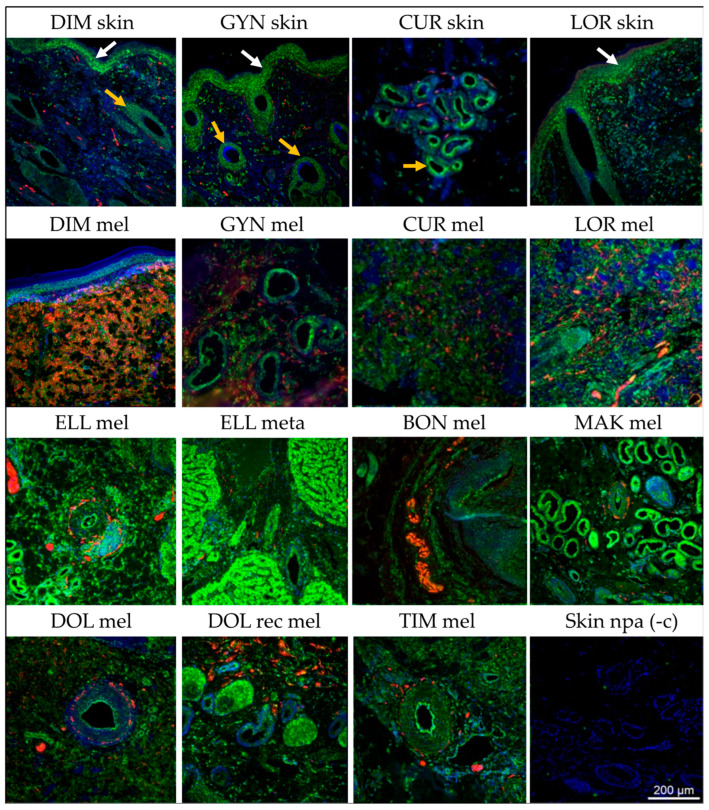
IF staining of normal skin and gray horse melanoma sections for MMP1 and S100. All intact skin and tumor sections (mel: cutaneous melanoma; rec mel: recurrent cutaneous melanoma; meta: melanoma liver metastasis) were co-stained for MMP1 (green signal) and S100 (red signal; nuclei were counterstained with DAPI (blue signal). The arrows in the top row point to hair shaft and endothelial cells (white) and glandular cells (yellow). Skin npa (-c): no-primary-antibody control. Images were taken using a fluorescence microscope (Axio Imager Z2, Zeiss, Jena, Germany) equipped with a highly sensitive camera (AxioCam MR R3, Zeiss, Jena, Germany).

To assess MMP1 expression on a protein level, sections from nine distinct melanoma and four normal skin samples were IF-labeled for MMP1 (green signal) and the melanoma marker S100 (red signal). Cell nuclei were counterstained with DAPI (blue signal). A no-primary-antibody control was included [Figure 3, skin npa (-c)] in all experiments. The results are presented in Figure 3. Grayscale images at higher resolutions are provided in Supplementary Materials, Figure S1.

In the normal skin obtained from melanoma-affected horses (Figure 3, top row), MMP1 expression (green signal) was exhibited by glandular cells (yellow arrows). The epithelial staining observed in several cases may indicate hair shaft and endothelial cells (white arrows). The selective staining for S100, as observed in the case of DIM skin, is likely exhibited by nerves.

In melanoma tissue, MMP1 expression was not restricted to glandular cells. The staining patterns suggested that cancer cells also expressed the enzyme. The signals were most pronounced in the melanoma tissue of TIM, CUR, LOR, and ELL. The MMP1 staining of melanoma sections of GYN and BON were difficult to interpret because of tissue denaturation, as reflected by unspecific DAPI staining (blue signal).

Except for the melanoma tissue of DIM that revealed intense co-staining for MMP1 and S100, the staining of the other tumor sections for S100 was less pronounced, or absent (ELL meta, i.e., ELL liver metastasis).

**Figure 4:**
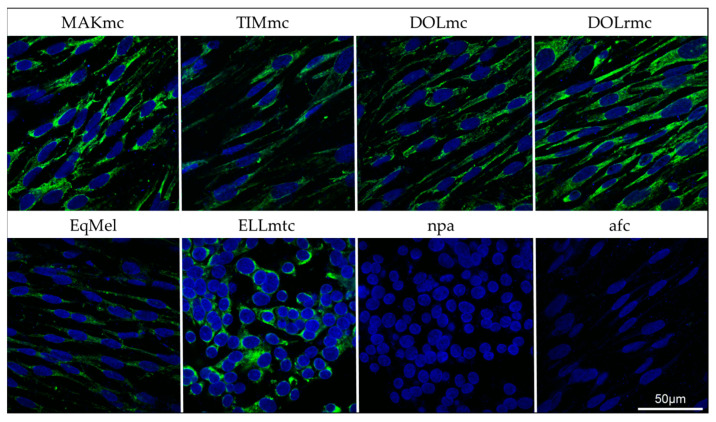
IF staining of equine melanoma cells for MMP1. Cells were stained for MMP1 (green cytoplasmic signal) and nuclei were counterstained with DAPI (blue signal). Upper-case designations: horse codes according to Table 2. mc: melanoma cells; rmc: melanoma cells from recurrent tumor; mtc: liver metastasis cells; EqMel: control melanoma cells; npa: no-primary-antibody control; afc: autofluorescence control. Images were taken using a confocal laser scanning microscope (CLSM 880, Zeiss, Jena, Germany).

To further confirm that MMP1 is specifically expressed by tumor cells, gray horse primary melanoma cells were established from five distinct lesions and subjected to IF staining for MMP1. EqMel served as control cells in the experiment. All primary cells’ preparations tested positive for MMP1 expression as shown in Figure 4. Overall, 100% of tumor cells exhibited cytoplasmic MMP1 expression irrespective of their origin. Staining was most pronounced in the case of tumor cells from MAK (MAKmc), the recurrent lesion of DOL (DOLrmc), and the liver metastasis cells of ELL (ELLmtc). The latter cells exhibited a round to polygonal morphology, whilst all other tumor cells were fusiform.

## 4. Discussion

In human MM, the expression of MPs is deregulated in the tumor and its microenvironment, and the pro- or anti-tumoral effect of most of these proteases is relatively well understood [5]. In equine melanoma, the pattern of MP expression has not been addressed so far. Herein, we report on the differential transcription of MPs in gray horse melanoma compared to intact skin tissue, and on the expression of MMP1 in gray horse melanoma tissue and primary cells established therefrom.

RNAseq was selected as the method of choice to establish the transcription profiles of equine cutaneous melanoma and normal equine skin. Overall, the study aimed at obtaining maximum information on the tumor-specific deregulation events affecting MPs and other genes potentially involved in the pathobiology of gray horse melanoma. In this context, the isolation of RNAseq-compatible RNA from tumor tissue proved difficult because 50 to virtually 100% of the tumor cells were highly pigmented (see Figure 2). As a result, only four tumor-derived mRNA isolates met the necessary quality criteria. Hence, the reduced number of samples subjected to RNAseq constituted a limiting factor of the study that had to be borne in mind when interpreting the results.

RNAseq revealed a series of deregulated MPs. The most significant findings included the transcriptional upregulation of MMP1 (125-fold), ADAM28, MMP9, and ADAM9 (5.2-fold, 4.4-fold, and 4.2-fold upregulated) in tumor tissue compared to normal skin. The pronounced upregulation of the MMP1 transcription according to RNAseq was further confirmed by MMP1 RT-qPCR from additional tumor and skin samples. Interestingly, comparative MMP1 RT-qPCR from primary melanoma cells (DOLmc) and normal skin tissue originating from the same individual revealed similar MMP1 transcription levels. This finding agrees with the observation that MMP1 is predominantly expressed by stromal fibroblasts in skin and at early hMM stages (RGP) [22].

The radial and vertical growth phases (RGP, VGP) of human MM are each associated with distinct MP expression patterns, emphasizing that hMM progression is driven by a concerted action of protumoral MPs, whilst anti-tumoral MPs remain inactive [7]. When grossly comparing the MP expression signatures of RGP and VGF hMM presented by Moro and colleagues [7] with herein reported MP transcription data, considerable differences became apparent: Apart from MMP1, other pro-tumoral MPs such as MMP2, MMP13, and MMP14 (MT-MMP1) [5,7,23,24,25,26,27,28,29] showed no deregulation of transcription in gray horse melanoma. In addition, the transcription of the protumoral MPs ADAM12 and ADAM15 was downregulated in the equine lesions compared to normal skin [7].

The enhanced transcription of MMP9 and ADAM9 observed in gray horse melanoma tissue is difficult to interpret, since the function of these proteases, at least in hMM, is site-dependent: There is evidence that MMP9 expression by hMM cells is associated with a pro-tumoral function, whereas stromal MMP9 expression seems to have an anti-tumoral effect [30,31]. Contrarily, tumor cell-derived ADAM9 confirmedly drives hMM progression, whilst stromal ADAM9 expression may have a protective function [7]. More work is necessary to determine the respective MP expression profiles of equine melanoma cells versus tumor-associated stromal cells to address the pro- or anti-tumor functions of these MPs in the horse.

To determine MMP1 expression on a protein level, ten skin lesion and one metastasis as well as four matching normal skin samples were comparatively subjected to IF-staining, with S100 serving as a melanoma marker. In normal skin, MMP1 staining signals were exhibited by glandular cells. This finding agrees with the evidence provided by The Human Protein Atlas (v23.proteinatlas.org). Epithelium also tested positive for MMP1. This finding is not surprising since human endothelial cells, hair cortex cells, and inner and outer root sheet cells are described to express the enzyme (v23.proteinatlas.org). In tumor tissue, the MMP1 signal was not restricted to said cell types, but also exhibited by other cells that likely corresponded to tumor cells. This was most evident in the case of DIM mel, where most cells consistently co-expressed MMP1 and S100. In all other cases, S100 co-expression was less pronounced, rendering the unequivocal identification of melanoma cells more challenging. The S100 gene family is the largest subfamily of EF-hand calcium-binding proteins and considered a reliable diagnostic marker in human melanoma and metastasis [32]. In 2004, we assessed gray horse melanoma for S100 expression by immunohistochemistry (IHC) using a polyclonal antibody recognizing the proteins’ α and β subunits. This resulted in the uniform detection of high S100 expression levels in 27/27 cutaneous gray horse melanomas [11]. In this study, lesions stained S100-positive to various extents, with signals ranging from intense (DIM mel) to absent (ELL meta). This observation could be attributable to the use of an anti-S100-A1 antibody that is α subunit-specific. Xiong and colleagues have recently studied the individual transcription pattern of each of the 25 S100 family gene members in human MM versus normal skin and correlated the obtained results with clinicopathological data. Overall, the transcription of individual S100 family members was not uniform and deregulated depending on the AJCC grade and the presence of lymphatic and distant metastases [32]. On these grounds, the use of the anti-S100-A1 antibody in our study may have failed to provide a complete picture on S100 upregulation in gray horse melanoma cells by IF.

To ascertain that MMP1 is specifically expressed by melanoma cells, primary cell cultures were established from four cutaneous lesions and a liver metastasis. The tumor cells derived from the skin lesions including the EqMel positive control cells were uniformly spindle-shaped, corresponding to a dedifferentiated phenotype that is also found in tumor tissue: Previous histological analyses of gray horse melanomas have revealed the intralesional presence of epitheloid, spindle-shaped, polyhedral, and balloon-shaped melanoma cells [11]. The primary cells originating from the liver metastasis (ELLmtc) displayed a polyhedral/round morphology indicative of a differentiated, hyperproliferative phenotype. This interpretation was also substantiated by cell-doubling times that were significantly shorter in the case of cells derived from the metastasis. All cells produced various levels of melanin as reflected by the color of the growth medium, with excessive pigment synthesis being noted in the case of ELLmtc.

MMP1 staining consistently yielded a cytoplasmic signal in 100% of the cultured melanoma cells. This finding unequivocally demonstrated that the zymogen is specifically produced by the tumor cells in gray horse melanoma. It is well established that MMP1 is a collagenase expressed by human stromal fibroblasts and cancer cells. The overexpression of MMP1 in hMM is associated with invasion and metastasis, as it degrades major components, i.e., fibrillar collagen types I, II, III, VII, and X, of the ECM. Moreover, MMP1 acts as a promoter of hMM neovascularization and the anti-apoptotic factor, thus contributing to hMM cell survival [25]. Although the exact pathobiological role of MMP1 in gray horse melanoma onset and progression remains to be elucidated, the herein reported overexpression of MMP1 in tumor tissue and melanoma cells derived therefrom suggests that this collagenase is actively involved in tumor cell migration and metastasis.

## 5. Conclusions

MPs have a major role in hMM progression and metastasis, and the radial and vertical tumor growth phases are each associated with a specific MP expression pattern that correlates with the stage and prognosis of disease [7]. Melanoma also affects horses, with individuals of gray coat color being particularly prone to acquire the disease. This predisposition is caused by a 4.6 kbp duplication of intron 6 within the synthaxin-17 gene that concurrently accounts for the gray phenotype and the frequent development of melanoma and vitiligo [12]. Unlike hMM, gray horse melanoma can remain almost quiescent for many years [9]. This phenomenon is subject to persistent controversies regarding the malignancy of disease and how lesions should be managed [33,34].

Given the accepted pathogenic role of MPs in hMM, and that two-thirds of melanoma-bearing gray horses exhibit metastatic disease at necropsy [34], we subjected tumor samples and normal skin to transcriptomic analysis. The RNA-seq approach revealed a deregulated MP transcription pattern in gray horse melanoma, with MMP1 showing the most significant upregulation in tumor tissue compared to intact skin. This finding was further confirmed by RT-qPCR from additional tumor and skin samples. Importantly, the overexpression of MMP1 was also demonstrated on a protein level. The tumor tissue sections tested consistently positive for MMP1 by IF staining. In addition, 100% of the primary tumor cells explanted from five distinct tumors exhibited cytoplasmic MMP1 expression. In sum, our findings suggest that MMP1 may have an active pathobiological role in gray horse melanoma similar to what is observed in hMM [7].

## Figures and Tables

**Table 1:** MP expression patterns in the RGP and VGP of human cutaneous MM as reviewed by Moro and colleagues [7].

MP Localization	MP Function	MPs with Deregulated Expression in Human MM	
		in the RGP	in the VGP
Tumor	protumoral	MMP1, 14, 15, 16, 21 ADAM9, 10	MMP1, 13, 14, 15, 16, 19 ADAM9, 10, 12, 17 ADAMTS18 (mutated)
	antitumoral	MMP7, 9, 12; ADAMTS5	-
	pro- and antitumoral	ADAM15, ADAMTS4	MMP9
Stroma	protumoral	MMP2, 9, 13; ADAM15	MMP1, 13
	antitumoral	ADAM9	ADAM9
	pro- and antitumoral	-	-

**Table 2:** Horse and sample specifications.

Code	Breed	Sex	Age	Short Disease Description	Samples Collected	Analyses
ARI	Warmblood	G	22	Four melanomas, thoracic region	One tumor	RNAseq
BON	Lipizzan horse	M	11	Multiple encapsulated melanomas under the tail root	One tumor	RT-qPCR, IF
CUR	Pony	G	>20	Single encapsulated melanoma under the tail root	Tumor	RT-qPCR, IF
					Intact skin	IF
DIM *	Trakehner mix	G	14	Multiple melanomas (anogenital, parotidal, ocular region, lips)	Anogenital tumor	RNAseq, IF
					Intact skin	
DOL	Icelandic horse	G	14	Multiple melanomas in the anogenital region; a recurrent lesion after surgical excision	Anogenital tumor	Cell culture, IF
					Recurrent tumor	Cell culture, IF
					Intact skin	RT-qPCR
ELL *	Trakehner mix	M	13	Large perianal tumor, organ metastases (spleen, liver, heart)	Perianal tumor	IF
					Liver metastasis	Cell culture, IF
GYN	Shagya Arabian	M	>20	Two encapsulated melanomas under the tail root	One tumor	RNAseq, IF
					Intact skin	
LOR	Welsh Mountain pony	G	19	Two encapsulated melanomas under the tail root	One tumor	RT-qPCR, IF
					Intact skin	
MAN	Lipizzan horse	M	14	Two encapsulated melanomas under the tail root	One tumor	RNAseq
MAK	Arabian horse	S	4	Single melanoma, inner thigh	Tumor	Cell culture, IF
TIM	Warmblood	M	15	Single encapsulated melanoma under the tail root	Tumor	Cell culture, IF
HAC	Andalusian horse	S	13	Melanoma-free	Intact skin	RNAseq
MIR	Lipizzan horse	M	16	Melanoma-free	Intact skin	RT-qPCR

G: gelding, M: mare; S: stallion; Age in years; * same familial origin; IF: immunofluorescence staining.

**Table 3:** Deregulated MMP transcription in gray horse melanoma according to RNAseq.

Gene	$\bar{x}$ Skin	$\bar{x}$ Mel	n-Fold Deregulation	*p*-Value	FDR
Transcription > 4-fold upregulated in gray horse melanoma compared to intact skin					
MMP1	353	44,222	125.2	<0.0001	<0.0001
ADAM28	83	435	5.2	<0.0001	<0.0001
MMP9	224	991	4.4	<0.0001	<0.0001
ADAM9	2300	9504	4.1	<0.0001	<0.0001
Transcription > 4-fold downregulated in gray horse melanoma compared to intact skin					
ADAM12	1662	277	6.0	<0.0001	<0.0001
MMP28	924	157	5.8	<0.0001	<0.0001
ADAMTS1	6568	1275	5.1	<0.0001	<0.0001
ADAM15	11,003	2374	4.6	<0.0001	<0.0001

**Table 4:** Relative MMP1 transcription in melanoma compared to intact skin.

Sample Type	Code	Target	Mean CT	ΔCT	Mean ΔCT	ΔΔCT	2^−ΔΔCT^
Melanoma	BON	RPS13	32.55	0.30	−0.61	−4.69	25.90
		MMP1	32.86				
	CUR	RPS13	32.89	1.36			
		MMP1	34.25				
	DIM	RPS13	30.02	0.52			
		MMP1	30.55				
	GYN	RPS13	35.69	−5.26			
		MMP1	30.43				
	LOR	RPS13	30.83	0.04			
		MMP1	30.87				
Intact skin	DIM	RPS13	30.56	6.32	4.09		
		MMP1	36.88				
	DOL	RPS13	36.73	−0.94			
		MMP1	35.78				
	LOR	RPS13	30.97	5.82			
		MMP1	36.79				
	MIR	RPS13	26.62	5.15			
		MMP1	31.77				

**Table 5:** Relative MMP1 transcription in DOLmc and intact skin originating from DOL.

Sample Type	Target	Mean CT	ΔCT	ΔΔCT	2^−ΔΔCT^
DOL melanoma cells	RPS13	25.697	0.122	1.07	0.48
	MMP1	25.819			
DOL intact skin	RPS13	36.73	−0.94		
	MMP1	35.78			

## Data Availability

All data are presented in this article and Supplementary Materials, Figure S1.

## Supplementary Materials

The following supporting information can be downloaded at: https://www.mdpi.com/article/10.3390/cells13110956/s1, Figure S1: Gray-scale images of MMP1-stained gray horse melanoma sections.
