# Supplementary material for: Deregulation of Metalloproteinase Expression in Gray Horse Melanoma Ex Vivo and In Vitro
Source: Cells. 2024 May 31;13(11):956. doi: 10.3390/cells13110956 (PMC11172212; doi:10.3390/cells13110956)
Supplement: Supplementary file 1 [file cells-13-00956-s001.zip › cells-3022283-supplementary.pdf]

Supplement 1

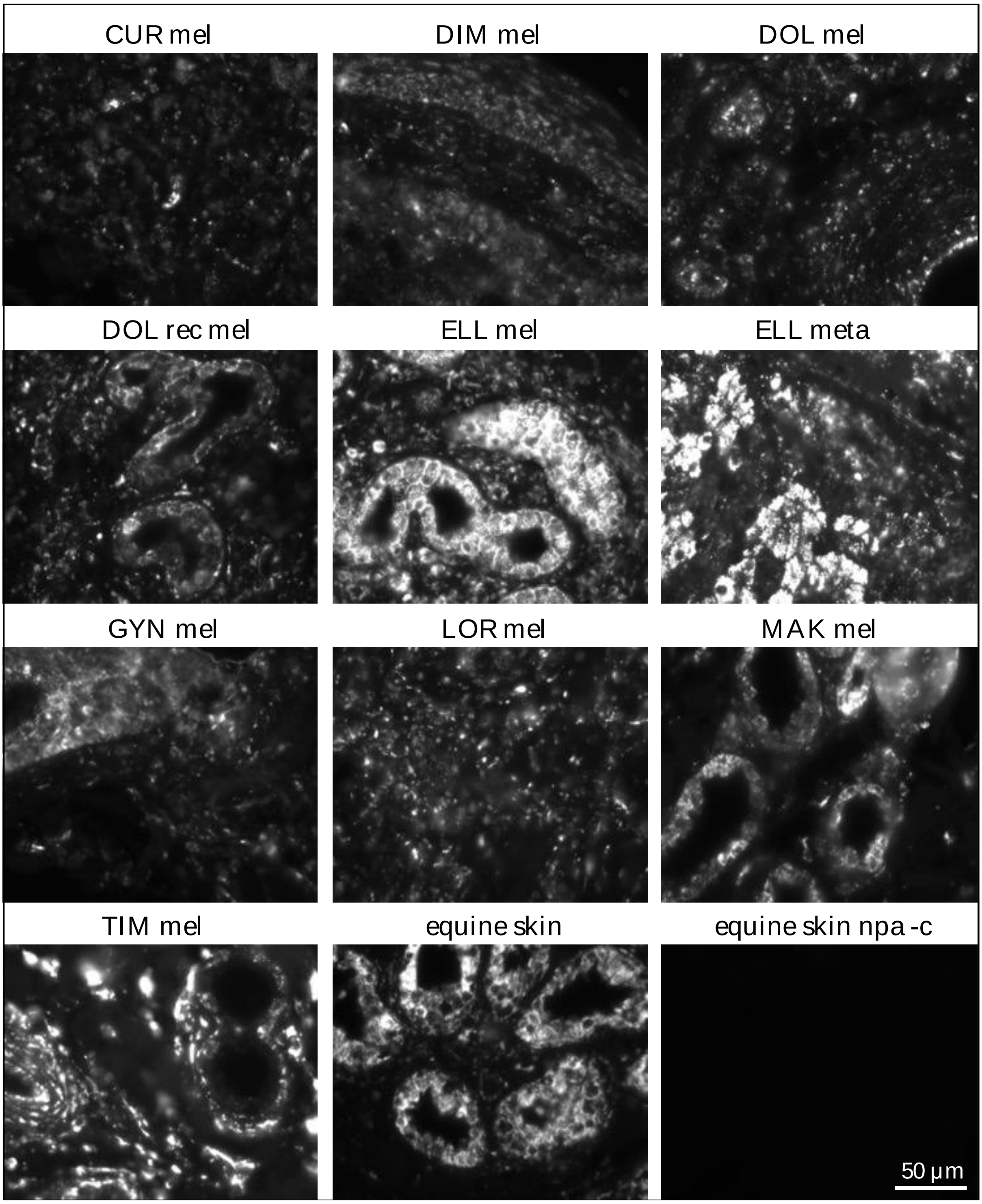

Figure S1: Grey-scale images of MMP1-stained grey-horse melanoma sections
In equine normal skin, MMP1 expression is confined to glandular cells. In the tumor sections, melanoma cells stain likewise positive for MMP1. Images were taken using an Epifluorescence Microscope Zeiss Axio Imager Z2 (Zeiss, Jena, Germany).
